# Benzothiazole Derivatives of Chitosan and Their Derived Nanoparticles: Synthesis and In Vitro and In Vivo Antibacterial Effects

**DOI:** 10.3390/polym15163469

**Published:** 2023-08-19

**Authors:** Tatsiana V. Shakola, Vasili V. Rubanik, Vasili V. Rubanik, Aleh V. Kurliuk, Anatoly A. Kirichuk, Alexander G. Tskhovrebov, Anton R. Egorov, Andreii S. Kritchenkov

**Affiliations:** 1 Faculty of Science, Peoples’ Friendship University of Russia (RUDN University), Miklukho-Maklaya St. 6, Moscow 117198, Russia; bychkovskaya-t@mail.ru (T.V.S.); kirichuk-aa@rudn.ru (A.A.K.); alexander.tskhovrebov@gmail.com (A.G.T.); 2 Department of General and Clinical Pharmacology, Vitebsk State Medical University, Frunze Av. 27, 210009 Vitebsk, Belarus; aleh.kurliuk@gmail.com; 3 Institute of Technical Acoustics NAS of Belarus, Ludnikova Prosp. 13, 210009 Vitebsk, Belarus; v.v.rubanik@tut.by (V.V.R.);

**Keywords:** chitosan, electrochemistry, nanoparticles, antibacterial activity, toxicity studies

## Abstract

In this work, we focused on synthesizing and assessing novel chitosan-based antibacterial polymers and their nanoparticles by incorporating benzothiazole substituents. The growing resistance to antibiotics has necessitated the search for alternative antimicrobial compounds. This study aimed to synthesize and evaluate chitosan-based polymers and nanoparticles with benzothiazole substituents for their antibacterial properties and toxicity. The benzothiazole derivatives of chitosan and their nanoparticles were synthesized through electrochemical coupling. The in vivo antibacterial efficacy was tested on white rats with induced peritonitis using a microbial suspension containing *S. aureus* and *E. coli*. Additionally, in vitro and in vivo toxicity assessments were conducted. The chitosan-based antibacterial systems showed significant in vivo antibacterial activity, surpassing that of unmodified chitosan and commercial antibiotics. Moreover, the toxicity studies revealed low toxicity levels of the synthesized derivatives, which did not differ significantly from native chitosan. The synthesized chitosan-based polymers and nanoparticles demonstrated potent antibacterial activity and low toxicity, highlighting their potential as effective alternatives to traditional antibiotics. Further investigations in pharmacology and preclinical trials are recommended to explore their application in clinical settings.

## 1. Introduction

Infectious diseases of bacterial etiology are one of the important causes of morbidity, disability, and even mortality [1,2,3]. Incredible progress in the etiotropic therapy of infectious diseases has been achieved due to the discovery of antibiotics [4,5]. However, over the past decades, attention has been increasingly paid to a number of problems associated with antibiotics, among which the most important are (i) the emergence of bacterial resistance to antibiotics and (ii) the undesirable side effects of antibiotics, including allergic reactions, as well as the effects caused by their general systemic toxicity [6,7,8,9,10,11]. In this regard, the search for new highly effective and safe antibacterial compounds is an important task in today’s medical chemistry, pharmacology, and related fields. Polymer chemistry often comes to the rescue in this matter [12]. Among polymers (including natural ones), several interesting systems with antibacterial activity have been described [13,14,15,16,17,18,19,20,21,22].

Chitosan is a biocompatible, biodegradable natural polymer that has inspired many researchers due to its biocompatibility, biodegradability, non-toxicity, and many other attractive biological activities, including antibacterial properties [23]. Based on chitosan, its attractive antibacterial derivatives were obtained, including triazolbetaine, betaine, azido, sulfur- and selenium-containing derivatives, and many others [24,25,26,27,28,29]. Advances in this field have been carefully reviewed in recent papers and books [30,31,32,33,34,35,36,37]. On the other hand, a few highly effective antibacterial low-toxicity compounds have been obtained based on the fused heterocyclic benzothiazole system [38,39,40,41,42]. These findings inspired us to synthesize benzothiazole derivatives of chitosan and study their antibacterial properties in the framework of our current work in the hope that the summation of the antibacterial effects of both chitosan and benzothiazole pharmacophore results in an increased antibacterial activity of the resulting polymers.

However, how to synthesize benzothiazole derivatives of chitosan with preparative simplicity? The fact is that in the electrochemistry of low-molecular-weight compounds, the coupling of aryl isothiocyanates with amines furnishing the formation of benzothiasoles has been thoroughly studied, and it is a completely classic issue. In the chemistry of chitosan (and, indeed, in the chemistry of polymers), this reaction has not yet been used. Moreover, this reaction is simple, convenient, and does not require harsh conditions. We decided to use the electrochemical combination of chitosan as an amine component with phenyl isothiocyanate to obtain benzothiazole derivatives of chitosan and then study their antibacterial effect and toxicity both in vitro and in vivo. We describe the work on this idea and discuss its results in the sections that follow below.

## 2. Materials and Methods

Chitosan (3.5 and 35 kDa; degree of acetylation 0.25), phenyl isothiocyanate, and acetonitrile were purchased from Aldrich, and alpha-MEM medium was purchased from Biolot, Ltd. (Beijing, China). Other chemicals and solvents were obtained from commercial sources and used as received.

The ^1^H NMR spectra were recorded on a Bruker spectrometer (Leipzig, Germany), operating at a frequency of 400 MHz.

High-resolution electrospray ionization mass spectrometry (positive ion mode) was carried out on a Bruker APEX-Qe ESI FT-ICR instrument (Madison, WI, USA), with CH_3_CN as a solvent.

The apparent hydrodynamic diameter and ζ-potential of the nanoparticles in water were estimated at room temperature (about 20 °C) using a instrument (Moscow, Russia) at λ = 659 nm and θ = 90°.

SEM images were obtained using an electron microscope JEOL JSM–6490LV (Tokyo, Japan), at 15kV, a SEM detector, and an electron beam size of 30 in high vacuum. The samples were coated with a layer of Pt in a JEOL auto-fine coater JFC–1600 (40 s at 40 mA, resulting in a nominal film thickness of 20 nm).

The viscosity of the chitosan solutions in 0.3 M NaCl/2% acetic acid was measured at 20 °C in an Ubbelohde viscometer. The intrinsic viscosity [η] of chitosan was calculated by extrapolation of the dependence ln(η_r_) × C^−1^ to an infinite dilution using the least-squares method. The viscosity-average molecular weight (MW) of the chitosan was calculated using the Mark–Kuhn–Houwink–Sakurada equation as follows: [η] = 3.41 × 10^–3^ × MW^1.02^ [43].

Chitosan derivatives were synthesized in an undivided electrochemical cell with a graphite rod anode (ϕ 6 mm) and a platinum plate cathode (15 mm × 15 mm × 0.3 mm) at a constant current of 7 mA and a current density of 11.7 mA/cm^2^. Chitosan 3.5 kDa (0.1 g; 0.5 mmol) was dissolved in water (2 mL), and 8 mL of acetonitrile with phenyl isothiocyanate (0.35, 0.6, or 1.35 equivs.) and *n*-Bu_4_NBF_4_ (1 equiv.) were added (solution A). Chitosan 35 kDa (0.1 g; 0.5 mmol) was dissolved in 1% acetic acid (10 mL), followed by precipitation with methanol (100 mL), centrifugation, and thorough washing of the chitosan with acetonitrile (100 mL). The washed chitosan was immediately dissolved in a mixture of water (2 mL) and acetonitrile (8 mL). Phenyl isothiocyanate (0.35, 0.6, or 1.35 equivs.) and *n*-Bu_4_NBF_4_ (1 equiv.) were added to the resultant solution (solution B). Solutions A or B were treated with a constant electrical current for 3 h. The formed polymers were precipitated with ethanol (100 mL), washed with ethanol (50 mL), and dried in air.

For the preparation of nanoparticles, the chitosan derivative **BCD-L-0.65** or **BCD-H-0.65** (1 mg) was dissolved in water (2 mL), and then sodium tripolyphosphate solution (0.5 mg/mL) was added (for volume, see Table 1). The rate of addition of the polyphosphate solution should be no more than 3 drops per minute, and intensive stirring is also necessary.

The antibacterial activity in vitro and in vivo (in white rats) and toxicity experiments were performed as described elsewhere [44,45]. The in vitro antibacterial activities of the chitosan-based systems were investigated using the agar-well diffusion method. The activity of the tested samples was studied against *S. aureus* (RCMB 010027) and *E. coli* (RCMB 010051). The activity was determined by measuring the diameter of the inhibition zone (in mm). Each inhibition zone was measured three times using a caliper to obtain an average value. Ampicillin and gentamicin were used as the antibacterial standard drugs.

Male Wistar rats of a 3-month-old line weighing 180–200 g were used in the work for evaluation of the in vivo antibacterial activity. All animals were kept in a vivarium under the conditions of a 24-h photo regime, controlled temperature (22 ± 2 °C), air humidity of 65 ± 10%, and free access to water and standard feed (granulated feed). The experiments were carried out in the first half of the day (10:00–13:00 Moscow time) in compliance with the rules for humane treatment of laboratory animals. During the implementation of this work, the general requirements of the European Convention Directive 2010/63/EU of 22 September 2010 were met. The middle third of the right half of the abdominal wall was shaved for the white male Wistar rats, after which the skin was treated with an alcoholic solution of iodine. A microbial mixture was used as an infectious agent, and the cavity in physiological saline was injected with 3 mL of a polymicrobial suspension consisting of the same amount of *S. aureus* and *E. coli* strains. After a total of 31 h after infection in the control groups, 200 μL of exudate was collected with a sterile syringe. In the experimental groups, rats were injected with a solution (0.8 mg per 300 µL) of the chitosan-based system, chitosan in the form of hydrochloride, ampicillin, or gentamicin, after 24 h. After a total of 7 h after treatment, 200 µL of exudate was taken. Each exudate obtained was diluted in physiological saline for an hour, and 6 10-fold dilutions of 100 μL were prepared, which were applied evenly on a Petri dish with meat-peptone agar. Colonies were counted 24 h after incubation in a thermostat at 37 °C. Subsequently, the colony-forming units (CFUs) were recalculated per 1 mL of exudate.

For the MTT viability test, the tested solutions were prepared by serial dilutions in an alpha-MEM culture medium. A 0.1 mL volume of each solution was added to a confluent monolayer of cells cultured in a 96-well plate. Cells HEK293 were incubated for 24 h at 37 °C in an atmosphere containing 5% CO_2_. The cells were washed twice with PBS, and then 0.1 mL of 3-(4,5-dimethylthiazolyl)-2,5-diphenyltetrazolium bromide (MTT; 0.5 μg/mL) in PBS was added and incubated for 4 h. The supernatant was then replaced with 0.1 mL of 96% ethanol, and the absorbance was measured at 535 nm.

For the primary assessment of the in vivo toxic effect, Wistar male rats of the 3-month-old line weighing 180–200 g were kept in a vivarium under the conditions of a 24-h photo regime, controlled temperature (22 ± 2 °C), air humidity of 65 ± 10%, and free access to water and standard feed (granulated feed). The experiments were carried out in the first half of the day (10:00–13:00 Moscow time) in compliance with the rules for humane treatment of laboratory animals. The animals were divided into experimental and control groups. For the experimental rats group, the middle third of the right half of the abdominal wall was shaved, the skin was treated with an alcoholic solution of iodine, and the rats were injected once with a solution (0.8 mg per 300 µL) of the chitosan-based system. From the moment of injection and over the course of 14 days, we assessed the behavior patterns of the animals (typical eating and drinking habits, unimpaired coordination, standard breathing rates and depths, and regular bowel movements). Additionally, we assessed the frequency of urination and the coloration of urine.

The statistical significance of differences between the samples was determined by a one-way analysis of variance (ANOVA). Upon performing ANOVA, the differences between the sample means were determined using Tukey’s post-hoc test. This was conducted at a significance level of *p* < 0.05 using the JMP 7 software (SAS Campus Drive; Cary, NC, USA).

## 3. Results and Discussion

### 3.1. Synthesis of Benzothiazole Chitosan Derivatives

**Scheme 1:**
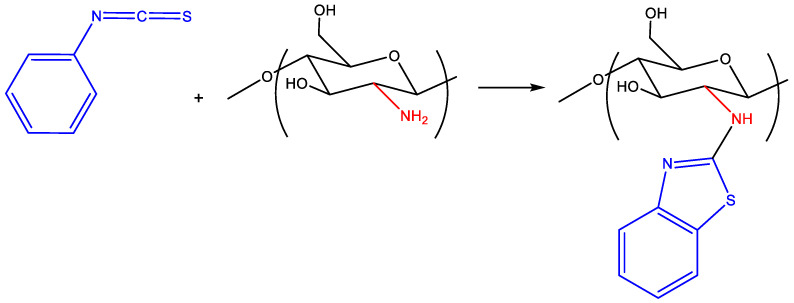
Electrochemical coupling of chitosan with phenyl isothiocyanate.

Benzothiazole derivatives of chitosan were obtained by a green electrochemical procedure, which has been described previously for coupling low-molecular-weight amines with aryl isothiocyanates [46]. In the current study, we used chitosan as a low-molecular-weight amine component in the mentioned electrochemical coupling (Scheme 1).

Since we decided to use the same electrochemical conditions as described for low-molecular-weight amines, we noticed that the authors of the previous paper [46] used aqueous acetonitrile as a solvent. However, the solubility of chitosan in a mixture of water and an organic solvent is extremely dependent on the molecular weight of the polymer. Therefore, at the first stage, we determined the molecular weight of chitosan, which makes it possible to obtain its solution in a mixture of acetonitrile/water, 8/2 (by volume). As a result of the optimization experiments, we found that the maximum average viscosity mass of chitosan, which allows it to easily dissolve in aqueous acetonitrile, is 35 kDa. We have found that to successfully dissolve the mentioned chitosan in aqueous acetonitrile, the following trick must be followed: Chitosan (35 kDa) should be dissolved in acetic acid, followed by precipitation with methanol and thorough washing of the precipitated chitosan with acetonitrile. We recommend that the washed chitosan be immediately dissolved in aqueous acetonitrile since, in dried form (or even freeze-dried), it loses its ability to dissolve in this solvent system. Higher-molecular-weight chitosans were unable to dissolve in aqueous acetonitrile, even using the described trick.

To evaluate the effect of the molecular weight of polymers on their biological properties in further experiments, we used not only 35 kDa chitosan but also 3.5 kDa chitosan to prepare benzothiazole derivatives of chitosan. Chitosan 3.5 kDa readily dissolves in water and does not precipitate when a 4-fold volume of acetonitrile is added. For convenience, hereinafter, chitosan with an average viscosity of 35 kDa will be referred to as high-molecular-weight chitosan and chitosan with an average viscosity of 3.5 kDa as low-molecular-weight chitosan.

The electrochemical interaction of chitosan with phenyl isocyanate proceeds smoothly under mild conditions and is completely finished in 3 h. The degree of substitution of the resultant polymers can be easily controlled by the molar ratio of the reactants. In all cases, a two-fold excess of the reagent should be used due to the partial electrochemically driven degradation of phenyl isocyanate [46]. Although previous work [46] states that it is desirable to carry out the electrochemical reaction under a stream of nitrogen, we have found that aerobic conditions do not reduce the yield of the product or the degree of substitution of the resulting polymers; however, the use of aerobic conditions is much more convenient preparatively. Also note that the isolation and purification of the resultant polymers are very simple and do not require a lengthy dialysis procedure. The resulting polymers can be easily precipitated with ethanol and washed with ethanol. After drying to a constant weight, they are purified.

Thus, the optimal conditions for electrochemical synthesis are as follows: (i) the molar ratios of the reactants chitosan and phenyl isocyanate are 1:0.35 (to synthetize polymers with a degree of substitution of 0.20), 1:0.6 (to achieve a degree of substitution of 0.30), and 1:1.35 (for synthesis of derivatives with a degree of substitution of 0.65); (ii) an undivided electrochemical cell with a graphite anode and a platinum cathode, an electrical current of 7 mA, and a current density of 11.7 mA/cm^2^; (iii) a temperature of ca. 20 °C and a reaction time of 3 h; and (iv) the presence of *n*-Bu_4_NBF_4_ as a phase-transfer catalyst.

All polymers derived from low-molecular-weight chitosan are soluble at neutral, alkaline, and acidic pH. The low-substituted polymers with a low degree of substitution (degree of substitution of 0.2) derived from high-molecular-weight chitosan are water-soluble only at acidic pH values, whereas moderate- (0.3) and high-substituted (0.65) polymers from high-molecular-weight chitosan proved also to be soluble at neutral pH.

**Figure 1:**
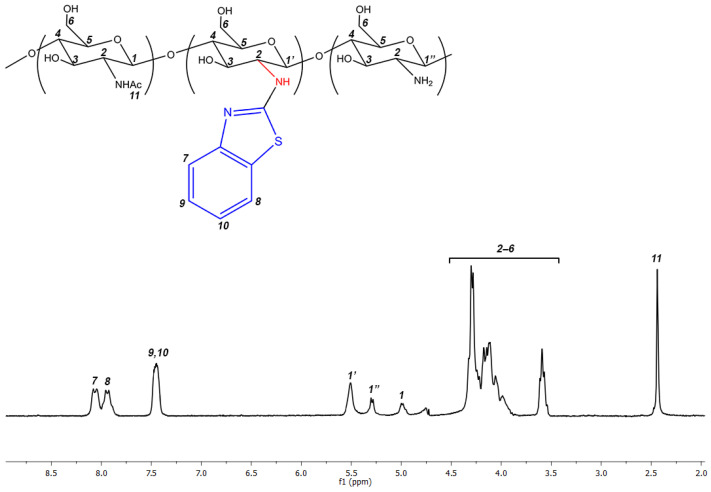
^1^H NMR spectrum of **BCD-H-0.65**.

The degree of substitution (DS) of the synthesized chitosan derivatives was established using ^1^H NMR spectroscopy data according to the following formula: DS = I(**1**′)/I(**1**,**1**′,**1**″) or DS = I(***Aromatic***)/4, provided that I(**1**,**1**′,**1**″) = 1, where I is the integral signal intensity of the corresponding protons. Selective *N*-substitution was proved by the equality of the value of DS, calculated by both above formulas. A typical ^1^H NMR spectrum of the obtained polymers with signal assignment and proton numbering is shown in Figure 1. The abbreviated names of the resultant polymers are as follows: **BCD-H-0.20**, **BCD-H-0.30**, and **BCD-H-0.65** or **BCD-L-0.20**, **BCD-L-0.30**, and **BCD-L-0.65,** where **BCD**—**b**enzothiazole **c**hitosan **d**erivative; **L**—**l**ow-molecular-weight chitosan; **H**—high-molecular-weight chitosan; 0.20, 0.30, and 0.65 are the corresponding degrees of substitution of the polymers.

Thus, we have introduced for the first time into the chemistry of chitosan (and into the chemistry of polymers in general) the electrochemical aryl isocyanate–amine coupling as an effective polymer-analogous transformation.

### 3.2. Preparation of Nanoparticles of Benzothiazole Chitosan Derivatives

There are many reports in the literature that the physicochemical, catalytic, or biological properties of polymers can be significantly improved by converting them into the form of nanoparticles [47]. A number of similar examples can be found in the chemistry of chitosan and its derivatives [28,45]. For example, nanoparticles of selenodiazole derivatives of chitosan are more successful catalysts for the oxidation of alcohols than the initial derivatives [24]. Nanoparticles of betaine derivatives of chitosan are much more effective as antibacterial compounds than starting polymers in their native form [29]. The same applies to a number of other chitosan derivatives, including diethylaminoethyl, trimethylaminobenzyl, pyridoxal, etc. [45].

To obtain polymer nanoparticles, a whole arsenal of modern methods can be used, including self-assembly, ultrasonic treatment, spray drying in an electric field, and ionic gelation [48]. All of the above approaches are widely used in the chemistry of chitosan, but, among them, ionic gelation has an important advantage: this approach is very simple and does not require special equipment derivatives [49]. For example, in our previous works [50,51], the ionic gelation method allowed us to obtain several chitosan-derived nanoparticles with a unimodal size distribution. Using this previous experience, in our current work, we used ionic gelation with sodium tripolyphosphate to prepare nanoparticles of highly substituted chitosan derivatives of different molecular weights. Looking ahead, it was highly substituted polymers that were chosen, since they are characterized by the best antibacterial properties.

The reaction of sodium tripolyphosphate and the benzothiazole derivative of chitosan is an electrostatic interaction that leads to the formation of a nanoparticle-shaped aggregate. This electrostatic interaction is realized due to the attraction forces between the tripolyphosphate anion and the protonated nitrogen atoms of the benzothiazole substituent of the chitosan derivative.

The hydrodynamic diameter of the obtained nanoparticles depends mainly on two factors: the molecular weight of the initial polymer and the amount of the added tripolyphosphate. Other things being equal, the addition of a larger amount of sodium tripolyphosphate first leads to a decrease in the hydrodynamic diameter of the resulting nanoparticles. The subsequent addition of tripolyphosphate, on the contrary, causes an increase in the size of the nanoparticles. Such observations have been repeatedly described in the literature and are quite typical for the preparation of nanoparticles by ionic gelation derivatives [49]. In addition, nanoparticles obtained from a high-molecular-weight chitosan derivative with the addition of the same amount of sodium tripolyphosphate are characterized by a larger size. As for the zeta potential, the addition of more and more sodium tripolyphosphate leads to a regular decrease in the zeta potential of the resulting nanoparticles.

The characteristics of nanoparticles of benzothiazole derivatives of chitosan obtained by ionic gelation are presented in Table 1.

**Figure 2:**
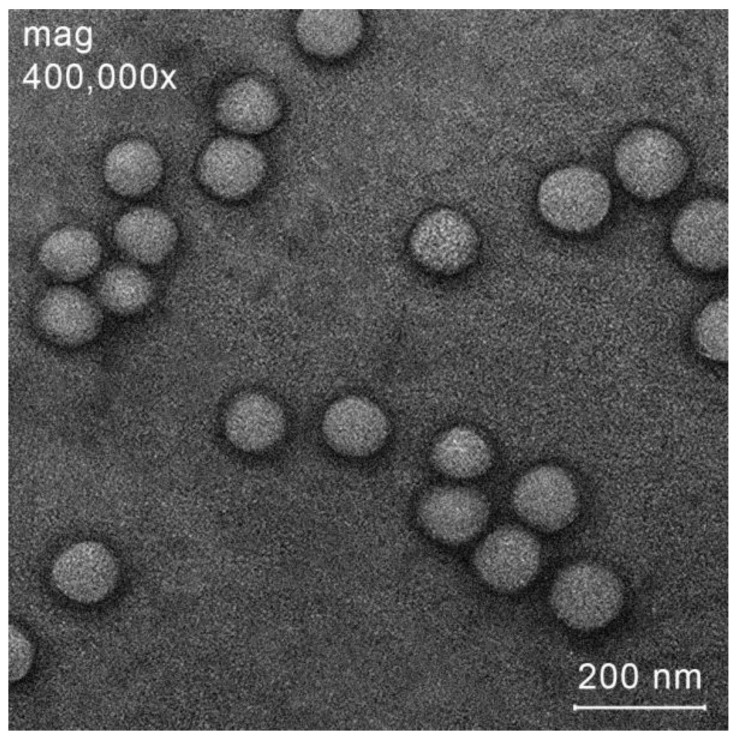
SEM image of NP-2-BCD-H-0.65.

The spherical shape, unimodal size distribution, and size of the resultant nanoparticles were also confirmed by scanning electron microscopy (SEM). A SEM image of the nanoparticles **NP-2-BCD-H-0.65** is presented in Figure 2.

### 3.3. Antibacterial Activity

#### 3.3.1. In Vitro Antibacterial Activity

Among a wide range of biological effects, including antioxidant, antitumor, immunomodulatory, anthelmintic, antileishmanial, anticonvulsant, and anti-inflammatory, the benzothiazole pharmacophore is also able to show strong antibacterial effects [38]. This circumstance inspired us to evaluate the antibacterial activity of the synthesized benzothiazole derivatives of chitosan and their nanoparticles. For a preliminary assessment of the antibacterial effects of chitosan derivatives, the agar diffusion method is usually used, which makes it possible to find the leaders for further testing [52].

In the current work, we used the agar-well diffusion test. This simple and convenient test is based on the inhibition of the growth of microorganisms around the well. Compounds with greater antibacterial activity provoke a larger zone of bacterial growth inhibition. Thus, by comparing the diameters of the inhibition zones, one can compare the antibacterial effects [53]. In this work, we compared the antibacterial activity of the synthesized systems with that of the starting chitosans and classical antibiotics (ampicillin and gentamicin) against the Gram-positive bacterium *S. aureus* and the Gram-negative bacterium *E. coli*. The results of the test are presented in Table 2.

The antibacterial effect of all tested systems is more pronounced than that of the starting chitosans. The activity of the original chitosan differs as follows: low-molecular-weight chitosan exhibits a greater antibacterial effect. However, for chitosan derivatives, the effect of the influence of molecular weight is practically leveled as follows: the antibacterial activity of chitosan derivatives does not depend on their molecular weight but strongly depends on their degree of substitution. An increase in the degree of substitution results in a dramatic increase in the antibacterial effect.

In certain cases, the conversion of polymers into the form of nanoparticles really contributes to an increase in antibacterial activity. However, this applies only to nanoparticles with the highest zeta potential and the smallest hydrodynamic diameter. Similar patterns have been described previously [45].

Thus, in each series (chitosans, benzothiazole polymers, and nanoparticles), we found the following leaders: low-molecular-weight chitosan (among chitosans), **BCD-L-0.65** and **BCD-H-0.65** (among polymers), and **NP-2-BCD-H-0.65** (among nanoparticles). These systems were involved in further in vitro experiments to study biological properties.

There are many interpretations of the mechanism of the antibacterial action of chitosan and its derivatives in the literature [32]. According to modern concepts, the main mechanism of the antibacterial action of chitosan is provided by the polycationic nature of its macromolecule [54]. The chitosan polycation is able to interact with high affinity with the anionic moieties of a microorganism’s cell surface [55,56]. This interaction causes a pronounced disruption of the ion channels, disruption of the processes of endocytosis and exocytosis, and, ultimately, provokes a rupture of the bacterial cell wall. The rupture of the cell wall is accompanied by the inevitable death of the microorganism [57].

Additional mechanisms of the antibacterial action of chitosan are also described in the literature. Chitosan is a natural polymer with extreme polydispersity (this is, in fact, a direct consequence of the methods of its production and isolation). Therefore, any sample of chitosan contains both high- and low-molecular-weight fractions [47]. Sufficiently small molecules of the chitosan polycation are able to penetrate into the bacterial cell and electrostatically connect anionic DNA molecules [58]. This results in the cessation of protein synthesis and, ultimately, the death of the bacterium. Another alternative mechanism involves the chelation of metal ions important for bacterial metabolism by chitosan. This leads either to death or to a slowdown in the growth of bacteria, as it provokes a number of metabolic disorders [59].

However, antibacterial derivatives of chitosan bear various substituents in their macromolecules, which are not typical for chitosan in its native form. Therefore, chitosan derivatives (especially highly substituted ones) may not have the same antibacterial mechanisms as starting chitosan. In this work, we tested whether elaborated chitosan derivatives and their nanoparticles have the ability to disrupt the integrity of the bacterial cell wall (the main mechanism of the antibacterial action of chitosan). We used the classical method based on the fact that bacteria with a damaged cell wall release content into the external environment, and this content intensively absorbs at 260 nm. Therefore, the greater the absorption at 260 nm, the more damaged bacteria are in the treated suspension [60].

**Figure 3:**
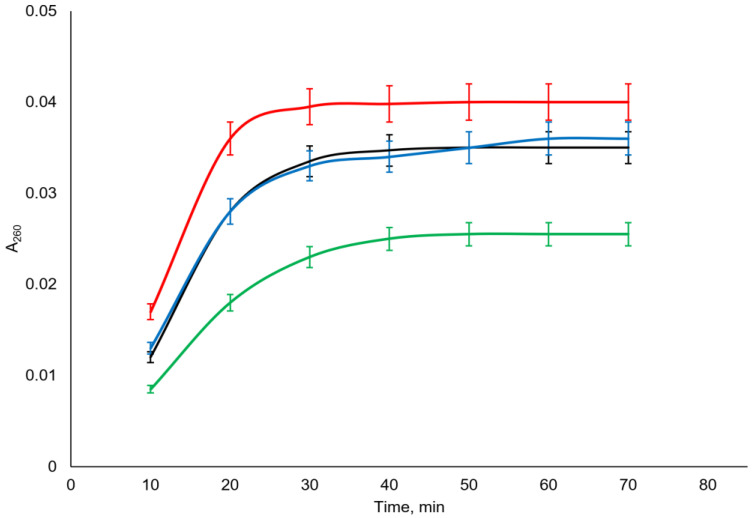
The release of the contents of bacterial cells that absorb at 260 nm from *E. coli* under treatment of bacterial suspension with low-molecular-weight chitosan (green), **BCD-L-0.65** (blue), **BCD-H-0.65** (black), and **NP-2-BCD-H-0.65** (red).

**Figure 4:**
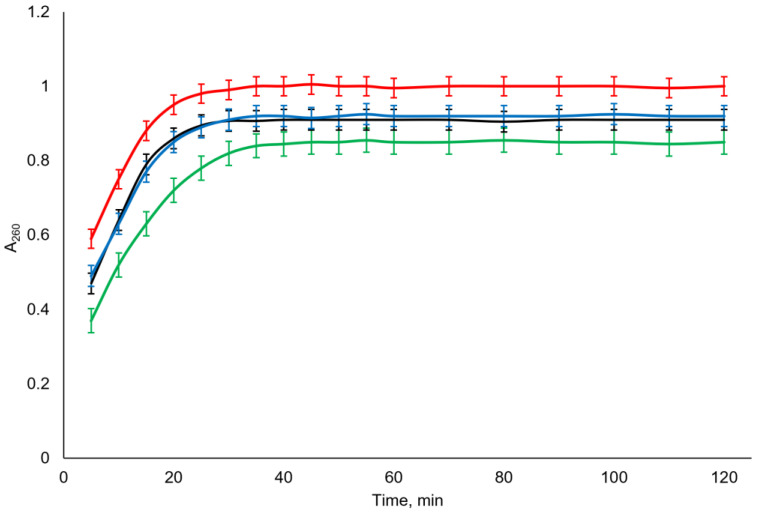
The release of the contents of bacterial cells that absorb at 260 nm from *S. aureus* under treatment of bacterial suspension with low-molecular-weight chitosan (green), **BCD-L-0.65** (blue), **BCD-H-0.65** (black), and **NP-2-BCD-H-0.65** (red).

We have treated bacterial suspensions with low-molecular-weight chitosan, **BCD-L-0.65**, **BCD-H-0.65,** and **NP-2-BCD-H-0.65**. The results of the experiments are presented in Figure 3 and Figure 4.

Firstly, all elaborated antibacterial systems (**BCD-L-0.65**, **BCD-H-0.65,** and **NP-2-BCD-H-0.65**) damage bacterial membranes to a much greater extent than the starting chitosan, since the bacterial suspension of *S. aureus* treated with these systems or *E. coli* absorbs light at 260 nm much more strongly. Secondly, the ability to damage the bacterial cell wall is almost the same for both of the tested polymers **BCD-L-0.65** and **BCD-H-0.65**. Thirdly, **NP-2-BCD-H-0.65** nanoparticles have proven to be the most effective cell wall pests for both *S. aureus* and *E. coli* bacteria. These nanoparticles not only damage bacteria to a greater extent but are also characterized by a much higher rate of inducing this effect.

#### 3.3.2. In Vivo Antibacterial Activity

Undoubtedly, the discovery of a highly active in vitro antibacterial polymer is a good success in the search for new antimicrobial compounds. However, this is only the beginning of a long journey, and further biological tests are needed, including in vivo ones. There are cases when highly active in vitro compounds, for some reason that is sometimes not entirely clear, do not show the necessary pharmacological activity in vivo. In this work, we evaluated the antibacterial effect of the **BCD-L-0.65**, **BCD-H-0.65,** and **NP-2-BCD-H-0.65** systems in vivo in white rats and compared this effect with that of the starting chitosan and the above-mentioned commercial antibiotics.

We studied the in vivo antibacterial activity in rats using model peritonitis; that is, the animals were specially infected by intraperitoneal injection of a microbial suspension containing *S. aureus* and *E. coli*. After six hours, the rats showed the typical attributes of peritonitis, which include listlessness, loss of appetite, frequent respiration, and puffiness. For the reference group, a 200 μL sample of exudate was collected one day post-infection. After twenty-four hours, the rats were injected with **BCD-L-0.65**, **BCD-H-0.65**, **NP-2-BCD-H-0.65**, chitosan, or the commercial antibiotics. Seven hours later, a 200 μL sample of exudate was collected from the treated rats. This exudate was mixed with a 0.9% sodium chloride solution (1000 μL). A total of 100 μL of the resulting mixture was inoculated on meat-peptone agar. After allowing it to incubate for 24 h at a temperature of 37 °C, the number of colonies was counted. The number of colony-forming units (CFUs) was then adjusted to represent the number per 1 mL of exudate.

Table 3 shows that the unmodified chitosan has the least in vivo effectiveness against the peritonitis-inducing bacteria. The antibiotics that were examined exhibited strong performance. The chitosan-based antibacterial systems that were elaborated (**BCD-L-0.65**, **BCD-H-0.65,** and **NP-2-BCD-H-0.65**) displayed the most potent in vivo antibacterial action, as evidenced by the scanty colony growth following exudate collection. The somewhat lesser performance of the market-available antibiotics could be attributed to their rapid expulsion from the system. In contrast, the removal of polymers and nanoparticles is a more arduous process, which could contribute to the enhanced antibacterial efficacy of the nanoparticles when compared to ampicillin and gentamicin [13].

### 3.4. Toxicity Study

Typically, natural polysaccharides, including chitosan, have dramatically low toxicity and are considered non-toxic biopolymers [60]. However, the introduction of a new substituent into the chitosan macromolecule can change the toxic characteristics of the resulting polymer [61]. In this work, we evaluated the toxicity of the obtained antibacterial systems based on chitosan (polymers and nanoparticles) in vitro and in vivo.

For the in vitro experiments, we used the so-called MTT test, which is very convenient for the initial assessment of toxicity. This test shows the number of surviving cells after exposure to the test substance [62]. The test results are presented in Table 4.

The results demonstrated that the in vitro toxicity of the synthesized species **BCD-L-0.65**, **BCD-H-0.65,** and **NP-2-BCD-H-0.65** does not differ from that of the starting chitosan. This allows us to consider the obtained antibacterial systems as non-toxic and to state that the introduction of the benzothiazole substituent into the chitosan backbone does not increase the toxicity of the initial polysaccharide.

The preliminary estimation of the in vivo toxic effects of **BCD-L-0.65**, **BCD-H-0.65,** and **NP-2-BCD-H-0.65** was performed by a previously published standard procedure [63]. We found that after injection of **BCD-L-0.65**, **BCD-H-0.65**, or **NP-2-BCD-H-0.65,** the animals’ overall health was not compromised. There were no observations of acute toxicity symptoms and no fatalities among the rats. During the in vivo experiment, the behavior patterns of the animals in the test group remained within the bounds of physiological normality (typical eating and drinking habits, unimpaired coordination, standard breathing rates and depths, and regular bowel movements). Additionally, the frequency of urination and the coloration of urine appeared to be normal.

Thus, in vitro and in vivo tests confirmed that the synthesized antibacterial chitosan-based systems **BCD-L-0.65**, **BCD-H-0.65,** and **NP-2-BCD-H-0.65** are non-toxic. At the same time, the authors are aware that the last test (primary estimation of an in vivo toxic effect) has limitations and is only a preliminary assessment. Further detailed elucidation of in vivo effects requires histological analysis, blood tests, and a fully extended cohort of preclinical trials.

## 4. Conclusions

The results of this work can be considered from the following few perspectives:

Firstly, we are the first in polymer chemistry to use an electrochemical coupling between aryl isothiocyanates as a polymer-analogous transformation. In this polymer-analogous transformation, the natural polymer chitosan played the role of an amine component. Using the new polymer-analogous transformation in the chemistry of chitosan, we synthesized new benzothiazole derivatives of chitosan and obtained their nanoparticles (using the ionic gelation method).

Secondly, we evaluated the antibacterial activity of the obtained chitosan-based systems (new polymers and their nanoparticles) and found in in vitro experiments that polymers with high degrees of substitution, regardless of molecular weight, exhibit the highest antibacterial activity. At the same time, nanoparticles of a highly substituted derivative of high-molecular-weight chitosan are characterized by an even greater antibacterial effect. These leading antibacterial systems have confirmed their high antibacterial activity in in vivo experiments. Their in vivo activity was even slightly better than that of commercial antibiotics.

Thirdly, both in vitro and in vivo experiments have demonstrated that elaborated chitosan-based antibacterial systems are practically non-toxic. Their toxicity does not differ from that of chitosan (which is a non-toxic biopolymer), and these data allow them to be considered compounds of the IV class of hazards (low-toxic substances).

Finally, these results are very interesting for further studies of the obtained polymer systems in pharmacology and preclinical trials. It is possible that these polymer-based systems will prove themselves in the clinic as a nature-based alternative to antibiotics.

## Figures and Tables

**Table 1:** Characterization of nanoparticles of benzothiazole derivatives of chitosan.

Sample	Mean Hydrodynamic Diameter, nm *	V(TPP), mL	Polydispersity Index *	ζ-Potential, mV *
**NP-1-BCD-L-0.65**	150 ± 5	0.3	0.15 ± 0.05	22.0 ± 0.7
**NP-2-BCD-L-0.65**	100 ± 4	0.8	0.12 ± 0.03	20.1 ± 0.4
**NP-3-BCD-L-0.65**	180 ± 5	1.1	0.10 ± 0.02	21.4 ± 0.6
**NP-4-BCD-L-0.65**	250 ± 6	1.4	0.10 ± 0.04	13.3 ± 0.3
**NP-5-BCD-L-0.65**	350 ± 7	2.2	0.14 ± 0.06	7.5 ± 0.2
**NP-1-BCD-H-0.65**	150 ± 4	0.2	0.19 ± 0.05	31.3 ± 0.2
**NP-2-BCD-H-0.65**	100 ± 6	0.5	0.13 ± 0.03	27.3 ± 0.1
**NP-3-BCD-H-0.65**	180 ± 2	0.9	0.13 ± 0.05	21.7 ± 0.5
**NP-4-BCD-H-0.65**	250 ± 4	1.1	0.17 ± 0.07	18.5 ± 0.4
**NP-5-BCD-H-0.65**	350 ± 3	2.0	0.11 ± 0.04	12.2 ± 0.5

* Mean value ± SD; *n* = 3.

**Table 2:** In vitro antibacterial activity of the benzothiazole derivatives and their nanoparticles.

Sample	Bacteria	
	*S. aureus*	*E. coli*
	Inhibition Zone, mm *	
**BCD-L-0.20**	14.2 ± 0.5	12.0 ± 0.5
**BCD-L-0.30**	18.5 ± 0.3	13.9 ± 0.4
**BCD-L-0.65**	21.3 ± 0.1	15.7 ± 0.3
**BCD-H-0.20**	14.4 ± 0.4	12.2 ± 0.2
**BCD-H-0.30**	18.2 ± 0.3	13.7 ± 0.5
**BCD-H-0.65**	21.3 ± 0.3	15.5 ± 0.2
**NP-1-BCD-L-0.65**	21.9 ± 0.5	16.1 ± 0.2
**NP-2-BCD-L-0.65**	23.5 ± 0.3	17.4 ± 0.3
**NP-3-BCD-L-0.65**	20.5 ± 0.4	15.0 ± 0.4
**NP-4-BCD-L-0.65**	19.1 ± 0.2	14.0 ± 0.3
**NP-5-BCD-L-0.65**	18.4 ± 0.4	13.4 ± 0.3
**NP-1-BCD-H-0.65**	23.2 ± 0.2	18.2 ± 0.5
**NP-2-BCD-H-0.65**	27.4 ± 0.5	19.3 ± 0.4
**NP-3-BCD-H-0.65**	22.5 ± 0.3	17.8 ± 0.3
**NP-4-BCD-H-0.65**	21.3 ± 0.7	15.3 ± 0.2
**NP-5-BCD-H-0.65**	19.8 ± 0.4	14.0 ± 0.4
**Chitosan (LMW)**	13.6 ± 0.2	10.9 ± 0.3
**Chitosan (HMW)**	12.1 ± 0.5	9.1 ± 0.5
**Ampicillin**	30.5 ± 0.4	─
**Gentamicin**	─	22.2 ± 0.5

* Mean value ± SD; *n* = 3.

**Table 3:** In vivo antibacterial activity of the elaborated antibacterial systems.

Sample	CFU/mL of Exudate (7 h after Injection or 31 h after Infection) *
Control without treatment (24 h after infection)	2833 ± 141
Chitosan	1885 ± 93
**BCD-L-0.65**	12 ± 1
**BCD-H-0.65**	14 ± 1
**NP-2-BCD-H-0.65**	8 ± 1
Ampicillin	78 ± 2
Gentamicin	82 ± 3

* Mean value ± SD; *n* = 3.

**Table 4:** Toxicity of the elaborated chitosan-based antibacterial systems.

Sample	Cell Viability (%) at Sample Concentration 10 μg/mL *	Cell Viability (%) at Sample Concentration 300 μg/mL *	Cell Viability (%) at Sample Concentration 1000 μg/mL *
Chitosan	100	95 ± 2	62 ± 4
**BCD-L-0.65**	100	98 ± 1	65 ± 3
**BCD-H-0.65**	100	96 ± 2	63 ± 5
**NP-2-BCD-H-0.65**	100	97 ± 3	63 ± 2

* Mean value ± SD; *n* = 3.

## Data Availability

Not applicable.
